# Power-Saving Design of Radio Frequency Identification Sensor Networks in Bus Seatbelt Monitoring Systems

**DOI:** 10.3390/s20205882

**Published:** 2020-10-17

**Authors:** Sitong Sun, Wen Yang, Wilson Wang

**Affiliations:** 1 School of Automation and Electronic Engineering, Qingdao University of Science and Technology, Qingdao 266000, China; 4018040035@mails.qust.edu.cn; 2 Department of Mechanical Engineering, Lakehead University, Thunder Bay, ON P7B 5E1, Canada; wilson.wang@Lakeheadu.ca

**Keywords:** RFID sensor networks, sensor node, bus seatbelt monitoring, low power design

## Abstract

Seatbelt state monitoring is important in intercity buses for passenger safety. This paper discusses the issues and challenges in power-saving design of radio frequency identification (RFID) sensor networks in bus seatbelt monitoring. A new design approach is proposed in this work for low-power layout and parameter setting in RFID sensor nodes in hardware and software design. A one-to-many pairing registration method is suggested between the concentrator and the seat nodes. Unlike using extra computer software to write seat identification (ID) into an integrated circuit (IC) card, the node ID in this project can be stored into the concentrator directly, which can reduce intermediate operations and reduce development costs. The effectiveness of the proposed low-power design approach is verified by some experimental tests.

## 1. Introduction

For intercity buses, all passengers must always fasten their seatbelts for safety because of regulations in China. A reliable monitoring system is critically needed to examine if all the passengers have fastened their seatbelts on the journey. An intercity bus usually has 50–60 seats, and thus a wireless sensor network can be applied in this monitoring application.

**Figure 1:**
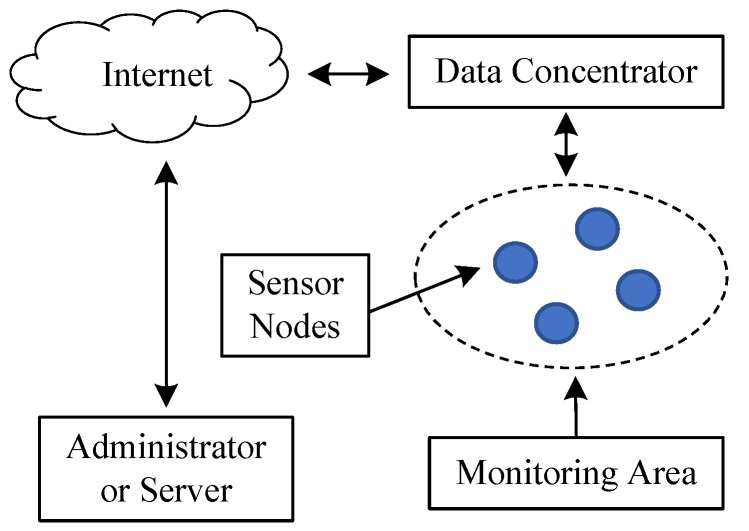
Topology of a wireless sensor network.

Wireless sensor networks have become widely used in industrial monitoring and control applications. A RFID sensor network includes both hardware and software. As illustrated in Figure 1, the hardware system consists of sensor nodes, concentrators, etc. The software includes control programs and communication protocols. RFID can transmit information of sensor nodes, including node ID and collected signals, to a higher level of the network through an internal radio-frequency (RF) circuitry. Multiple sensor nodes transmit data to the concentrator that can process and transmit the result to a background server through a base station and the internet. The field data collected by sensor nodes can establish background database for big data mining, remote monitoring, and analysis.

The power in a sensor node is usually provided by batteries. A battery has limited life capacity; but because of the related regulations, the battery lifetime must meet specific requirements (e.g., three years) in this monitoring application. It is thus a critical issue in design to reduce the power consumption of the sensor networks.

**Figure 2:**
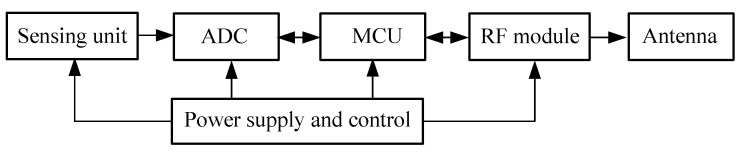
An active RFID sensor network node model.

A sensor node is usually composed of a sensing unit, an analog-to-digital (ADC) converter, a microcontroller unit (MCU), a RF module, a transmitting antenna, a power supply module, etc. As illustrated in Figure 2, firstly, the sensing unit measures physical parameters of the object. After appropriate pre-processing, the analog signal is converted into a digital counterpart by an ADC converter. By the communication protocol, the MCU transmits the collected data and the node ID to the concentrator wirelessly. The power supply must ensure the normal operation of each module.

To extend the service life, the nodes should be set to some sleep mode to reduce power consumption [1]. In hardware design, power can be reduced by setting proper I/O configuration and reducing the single-hop communication distance [2]. For example, the combinational logic circuit can be used to reduce the power consumption under the off-mode [3]. Low-power RF transceivers (e.g., 130 nm at 1 V) were used in [4] for wireless sensor networks, where the power oscillator (class E) is used. In paper [5], up to 80% standby energy could be saved using a battery management system by changing battery configuration and using voltage level shifters. An RF energy harvesting system was used in [6,7] to extend the life of wireless sensor nodes.

On the other hand, from a software perspective, frequency regulation can be used to adjust the central processing unit (CPU) clock frequency to save power [8,9]. The Contiki operating system was suggested in [10] to reduce the power consumption from the software perspective. A hybrid polling approach was proposed in [11] to reduce the number of frames transmitted by the router node. In paper [12], a quantization bit number was selected to optimize energy allocation as well as to reduce the error of the reconstructed fusion center. In some specific applications such as temperature measurement and monitoring, power can be saved by using three clocks to trigger interruption to turn on and off the custom control system [13]. In paper [14], a power consumption model was built by calculating power flow using a periodic algorithm. An electronic nose system was proposed in [15,16] to monitor gas temperature, concentration, and mixtures, and to predict the battery service life. Principal component analysis and linear discriminant algorithm were explored in [17] by using a decision tree and k-nearest neighbors. There were also some application examples in the literature for power saving, for example, in healthcare [18,19], airport logistics [20], MICa2 and n740 sensors [21]. However, these aforementioned strategies could bury the nodes’ ID and characteristics in communication, which will degrade power-saving control and influence advanced analysis.

Currently, most of the available bus seatbelt monitoring systems use state perception devices to measure the connection states [22,23]. The monitoring information about the seatbelt connection is sent to a dashboard for the driver [24]. Usually the driver provides a vocal warning to the passengers who have not fastened their seatbelts. However, these state perception systems could provide false warnings to the driver for those empty seats and when a seatbelt is re-connected [25]. In addition, these monitoring systems are not networked with the bus monitoring centers [26].

To tackle the aforementioned problems, the objective of this work is to develop and provide a guideline for low-power design of the RFID networks and a bus seatbelt monitoring system. A new design method is proposed for a low-power layout and parameter setting in RFID sensor nodes in hardware and software design. A one-to-many pairing registration method is proposed to match the concentrator and the seat nodes. Unlike using an extra computer software to write seat ID into an IC card, the node ID in this project can be stored into the concentrator directly, which can simplify operations and reduce development costs. The effectiveness of the developed seatbelt monitoring system was verified by experiments.

The remainder of this paper is organized as follows. The hardware power-saving design is discussed in Section 2. The software power-saving strategies are discussed in Section 3. The effectiveness of the proposed techniques is tested in Section 4, and some concluding remarks are summarized in Section 5.

## 2. Power-Saving Design in Hardware Systems

### 2.1. Overview

**Figure 3:**
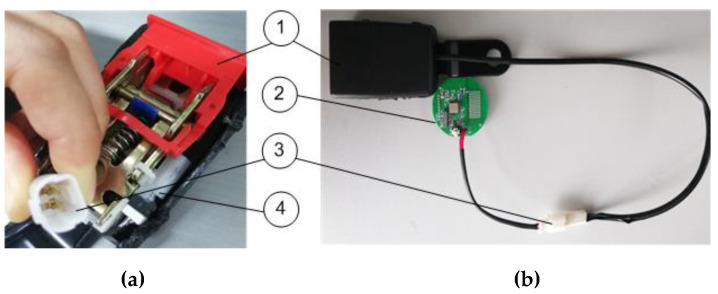
(**a**) A seatbelt buckle; (**b**) the measurement system: (1) buckle joint, (2) sensor node, (3) connector between the sensor node and the buckle, (4) trigger switch.

Figure 3 shows the sensor node we developed for the seatbelt monitoring system. It used RF 433 MHz ultra high-frequency (UHF) transmission circuit wireless communication. It could detect the connection status of each seatbelt. If the buckle was connected, the trigger switch would close, and then, power would be supplied to that node that will transmit data and belt-connection state to the concentrator.

**Figure 4:**
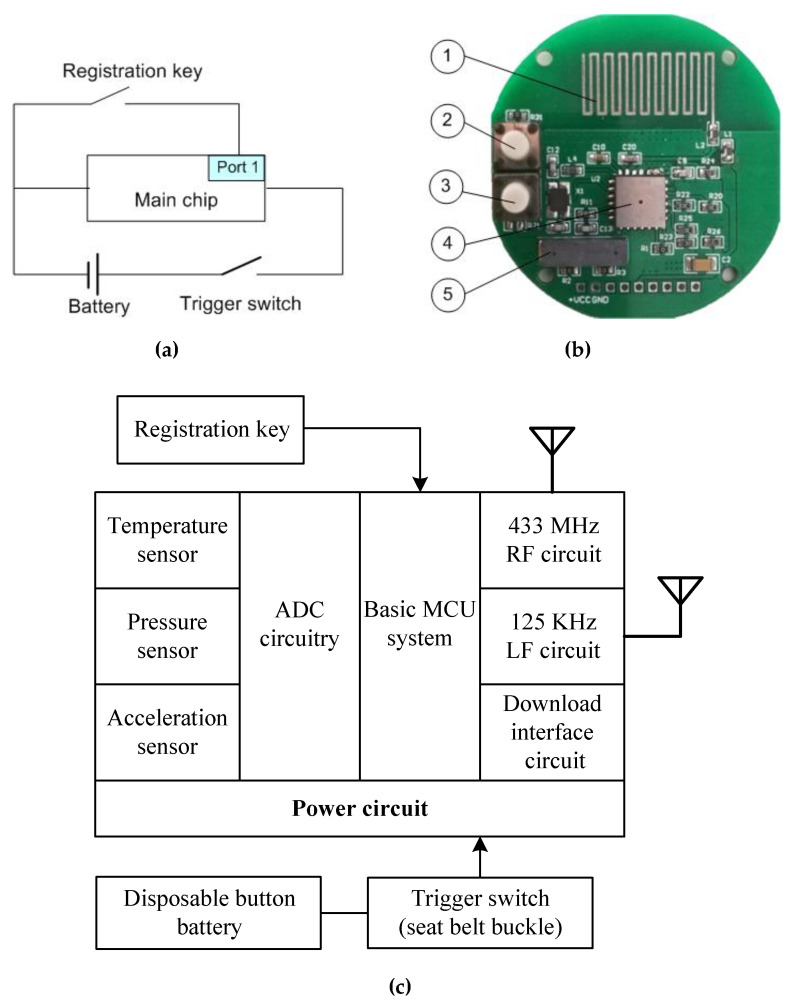
(**a**) The node schematic. (**b**) Node PCB diagram: (1) the RF antenna, (2) the trigger switch, (3) the registration key, (4) the main chip, (5) a 125 KHz low-frequency burning inductor. (**c**) Node internal structure.

The hardware was designed using Altium Designer 16.0. Figure 4 shows the node schematic and its printed circuit board (PCB) circuitry. The seat number was differentiated by using a registration key between the power supply pole and an I/O port of main chip (i.e., port 1). If a sensor node was to be registered with a seatbelt in the bus, the registration key should be pressed before the node is powered on, and then the node registration information is sent out. If the data counter in the concentrator adds one, the registration is successful.

Although the design of a 125-KHz low-frequency (LF) circuit could be a registration method, it required an extra software program to write vehicle information and seat IF into an IC card for the concentrator. To simplify implementation, a simple one-to-many registration method was suggested in this work. Figure 4c shows the node internal structure. The basic MCU is Freescale S08, which stores the node’s registration information. If the seatbelt is fastened, the power circuit is on. The registration key should be pressed before the belt is buckled, and then the node will send out its registration information. A 125-KHz burning inductor (B82450A7204A000) in Figure 4b was used to measure data and send them to the LF receiver. The receiver then checked the signal agreement, compared the ID information, and read the related data into the MCU. An antenna was designed to match transmission circuit impedance, which had a length of 17.2 cm according to $\frac{c}{4f}$, where *c* is the speed of light and *f* is the transmitting frequency (433 MHz in this case).

**Figure 5:**
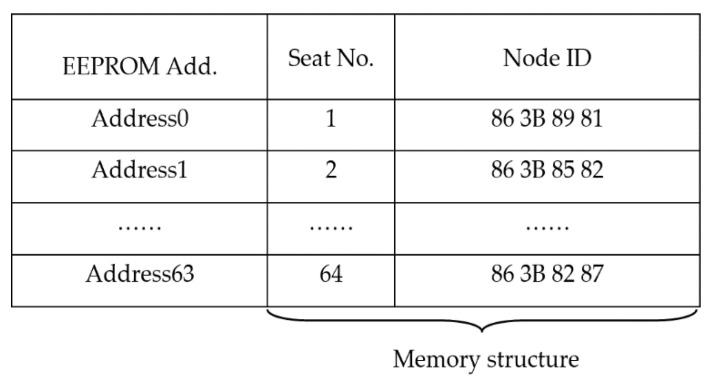
The structure of the concentrator EEPROM data storage.

The node transmitted data (e.g., node ID, cyclic redundancy check) to the concentrator. The related information was stored in the electrically erasable programmable read-only memory (EEPROM), as illustrated in Figure 5. The seat number was stored in the fixed EEPROM address of the concentrator. The node ID registry can be stored in the random-access memory (RAM) to reduce EEPROM reading/writing load and to improve processing speed. With the node ID and seat number, the concentrator can process the stored data and information.

The node operations were undertaken by the following procedures:

(1) Node Registration:
  - Turn concentrator power on.
  - Press “plus” key and make the data counter (i.e., digital tube) display “01”.
  - Press the node registration key and then fasten the seat safety belt (i.e., power on) to make node transmit registration ID.
  - If the data counter in the concentrator adds one to “02”, the node registration is successful.
  - Repeat these procedures until all of the 64-seatbelt node ID registration is completed.
  - Press the “reset key” to enter into normal mode.
(2) Normal Mode Control:
  - When the data counter displays “00”, the concentrator enters into its normal mode.
  - In the normal mode, the node will transmit seatbelt state data when it is buckled. The concentrator will receive the state data and check state data integrity.
  - If the state data belongs to this bus, it will be sent to RF controller. Otherwise, it will be cleared.
(3) Node Replacement:
  - Adjust the data counter to match the seat number if modification is required.
  - Press the new node registration key, and then fasten the seatbelt to transmit registration ID.
  - If the data counter in the concentrator adds one, the replacement of the new node is completed.
  - Press “Reset” key to enter into the normal mode.

### 2.2. Low-Power Design in Hardware Systems

**Figure 6:**
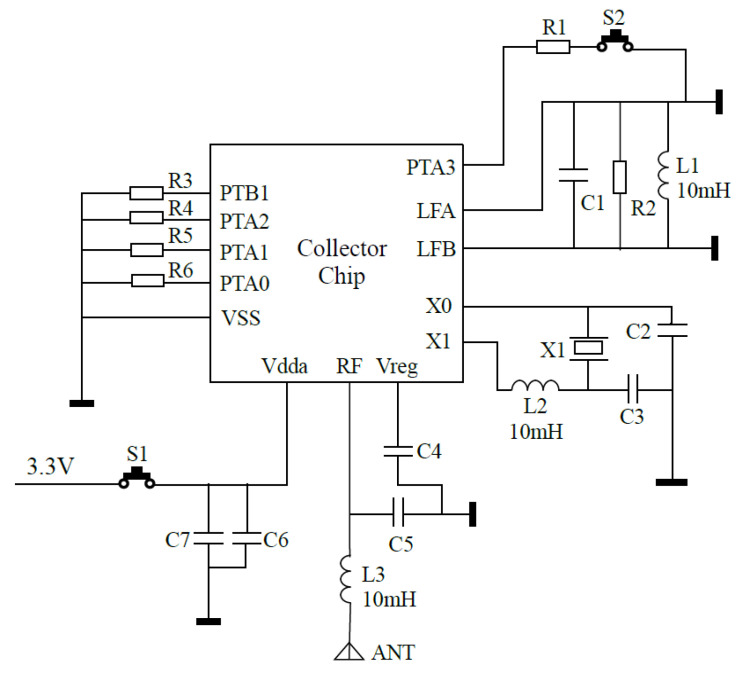
Node circuit schematic diagram.

The power saving in hardware involves integrated circuit (IC) design and peripheral circuit design. Figure 6 shows a node circuit schematic; the related power saving strategies will be briefly discussed next.

#### 2.2.1. Use of Low-Power Devices

It is useful in selecting high-input impedance and high-static resistance devices, as well as low-dynamic operation power devices to reduce power consumption. For example, preference was given to use complementary metal oxide semiconductor (CMOS) devices, followed by the complementary high-performance metal oxide semiconductors (CHMOS), high-performance metal oxide semiconductors (HMOs), and transistor-transistor logic (TTL) chips [27], and functional integrated low-power chips. In this seatbelt monitoring system, the low-power MCU of FXTH870902DT1 was used in each sensor node, as shown in Figure 6.

#### 2.2.2. Low-Power Supply Control

**Figure 7:**
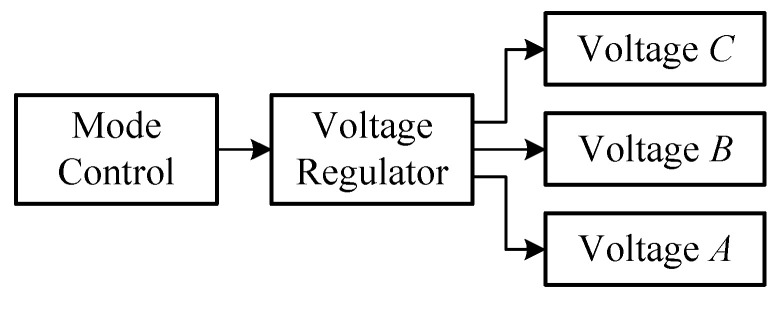
Specific voltage zone distribution layout.

In this seatbelt monitoring system, each sensor node power supply circuitry was divided into three zones of *A*, *B*, and *C*, as illustrated in Figure 7. Zone *A* had a 3-V power supply for the MCU system; zone *B* had 2.3 V of power for sensor nodes, and zone *C* had 1.8 V of power for the RF. If an area does not need to work over a certain period of time, the power supply to that area can be temporarily turned off. When it turns to work, the battery power can be activated to that area by software. For example, if a “0” is given to the related I/O port, the power supply to that partition is on, and vice versa.

#### 2.2.3. Low-Power Frequency Control

**Figure 8:**
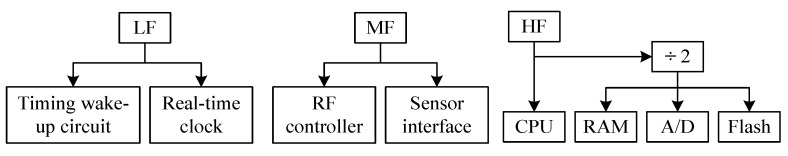
Clock frequency distribution diagram. LF—low frequency, MF—middle frequency, HF—high frequency.

The clock frequency of the system was divided into three ranges: LF of 1 KHz, middle-frequency (MF) of 125 KHz, and high-frequency (HF) of 8 MHz, as illustrated in Figure 8. In general, the LF clock circuit takes much less power than using the HF clock circuit, because HF operation takes more dynamic conversions during a unit time. But in most cases, the LF clock takes longer time than a HF clock in executing the same task, which will take higher power consumption. The sensor node in our developed seatbelt monitoring system was not always in its working state. Therefore different clock frequency in different working modes were used to reduce power consumption. For example, HF clock was used to complete the task of data acquisition and transmission. The LF clock was applied in non-working mode to reduce dynamic power consumption. In implementation, the sleep mode was divided into three states: sleep receiving mode, general sleep mode, and deep sleep mode. The node clock distribution of different modes is summarized in Table 1.

## 3. Power-Saving Design of the Software System

Under the condition of reliable communication, power consumption can be further reduced by using proper software control schemes. A flexible software control strategy will be suggested in this section to minimize power consumption in communication mode, node transmission cycle, duration of transmission, and sleep mode selection, as discussed below. CodeWarrior v10.7 is used to develop software programs [28].

### 3.1. Selection of Node Communication Mode

**Figure 9:**
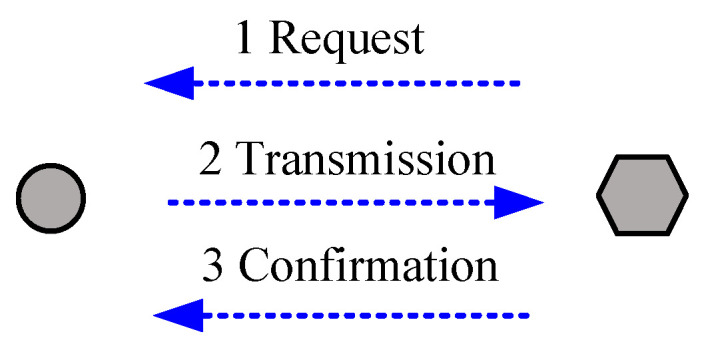
Passive node transmission mode.

The transmission communication in a sensor network can be undertaken in either an active mode or a passive mode. Figure 9 shows the schematic of a passive transmission mode, in which the node is usually in a sleep mode, but the concentrator is in its working state. Thus, if there is no data request from the concentrator or the host computer, the node remains in its receiving state or its receiving circuit does not go to sleep.

In using the active node sending mode, once the data preparation is completed, the node sends data out actively in a specific transmission cycle. When no data is to be transmitted, the circuit will turn to a sleep mode, which will be woken up regularly to check if there is data transmission command. Therefore, the power consumption of the active mode is lower than that of the passive mode, which will be used in this project.

**Figure 10:**
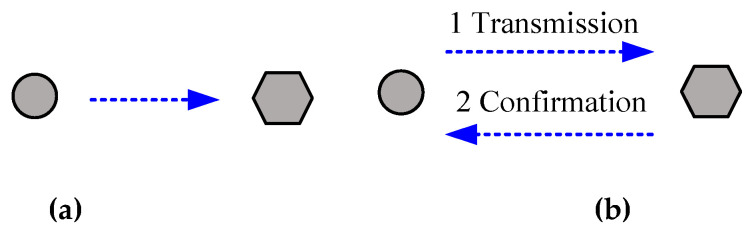
Active node transmission modes: (**a**) open-loop mode, (**b**) closed-loop mode.

As illustrated in Figure 10, an active node transmission mode includes open-loop active transmission and closed-loop active transmission. The node of the open-loop mode cannot realize whether or not its information is received by the concentrator. In the closed-loop mode, the concentrator generates a reception signal, and then the node will go to a sleep mode when there is no data to be transmitted.

**Figure 11:**
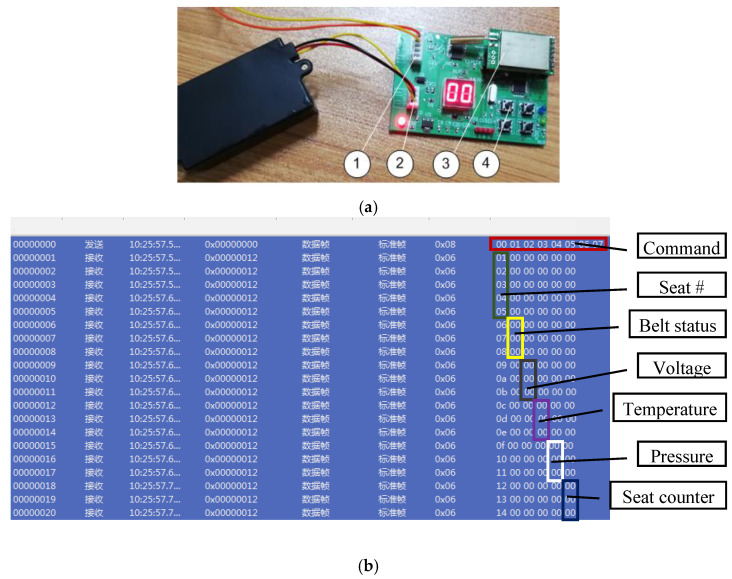
(**a**) The developed concentrator system: (1) controller area network (CAN) output, (2) power supply input, (3) 433 MHz wireless module, (4) registration keys. (**b**) The initial monitoring interface, where the data will come from the concentrator’s CAN output circuit.

Figure 11a shows our developed seatbelt monitoring system. It used the open-loop mode because the sensor nodes are the same type and do not need to be polled. The controller area network (CAN) protocol was used to ensure that information was received by the concentrator correctly. The concentrator in Figure 11a had four keys: the first two keys were for “plus” and “subtract”. When a certain registration node is broken or its battery is wearing out, the concentrator should adjust that node’s sequence number and make a new node registration as discussed in Section 2.1. The third key was to check node registration information by a CAN output function and then reset data counter to “00”. The fourth key was to check all seatbelt buckle statuses.

Once a request command is received, the CAN will read the state information in real-time, including the seat number, belt status, voltage value, temperature, pressure, and seat counter. Figure 11b shows the monitoring interface in its initial state. It will monitor the temperature and pressure to provide early warnings of possible dangerous conditions in an intercity bus.

### 3.2. Sending Period of the Node

**Figure 12:**
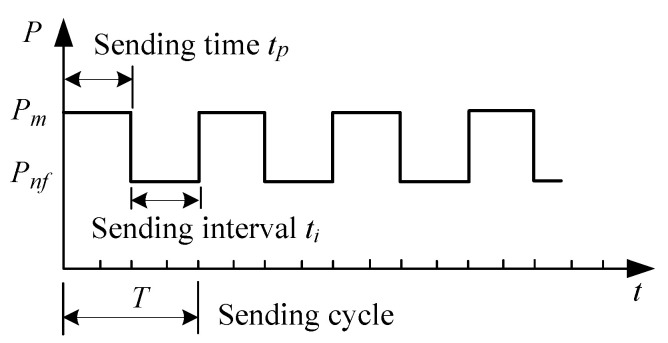
Transmission period model of nodes.

The transmission period refers to the time that the node takes to transmit data to the concentrator [29], including the sending time $t_{p}$ and sending interval $t_{i}$. As shown in Figure 12, the average node power $P_{avg}$ in mWh will be

(1) $$P_{avg}=P_{nf}+(P_{m}-P_{nf})\times \frac{t_{p}}{T}$$

where $P_{nf}$ is the power during the standby state; $P_{m}$ is the power taken during transmission, which must be high enough to ensure the reliable communication in transmitting data without missing data or slowing down the transmission rate.

$P_{nf}$ should be as low as possible to reduce power consumption. If $P_{nf}$ = 0, $P_{avg}$ will be the minimum, or

(2) $$P_{avg}=P_{m}\times \frac{t_{p}}{T}$$

Therefore, when $P_{m}$ and $P_{nf}$ are determined, the power consumption can be reduced by decreasing $t_{p}$ or increasing *T*. The sending time *T* is determined by the frequency of data transmission (9600 bit/sec in this case). Correspondingly the following software control methods will be proposed in different scenarios.

#### 3.2.1. Real-Time Transmission State

When the sensor, A/D converter, MCU, and transmission circuit are all in their operating state, the power consumption is the maximum. The life cycle of the node depends on its available power source [21]. In this case, the node should not be powered by a low capacity battery. It can use a wired power supply, instead. In our software program, a cyclic function was used to make measurement and transmission.

#### 3.2.2. Periodic Transmission State

The transmission can be in the form of continuous or periodic mode, depending on applications. Many applications do not require continuous data transmission and confirmation, such as human body temperature monitoring, chemical composition measurement, and animal position tracking [29]. According to Equations (1) and (2), the power can be reduced by increasing cycle *T* to extend the duration with no transmission. If the standby power consumption is not taken into account, for a given $t_{p}$, transmission efficiency will decrease as the transmission period increases. For example, in this seatbelt monitoring system, we have selected periodic transmission state by setting periodic wake-up functions.

#### 3.2.3. Determination of Transmission Duration $t_{p}$

**Figure 13:**
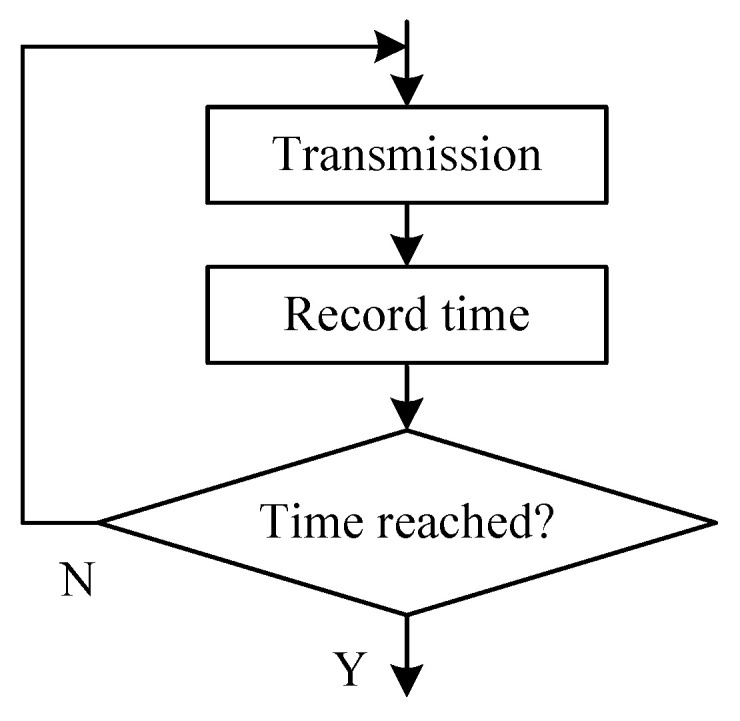
Processing flow chart of the open-loop active transmission mode.

It is unnecessary to repeat the data transmission process if the data has been received successfully by the concentrator [27]. However, it is unknown whether the reception is successful in an open-loop mode. For the sake of reliability, it is better to properly increase the transmission time according to transmission distance. But the increase of the transmission time will take more power consumption. Figure 13 shows the flow chart of an active open-loop transmission mode. If the reception is successful, the node will exit transmission mode and go to the standby mode. Otherwise the process is repeated until the specified time of transmission is reached.

**Figure 14:**
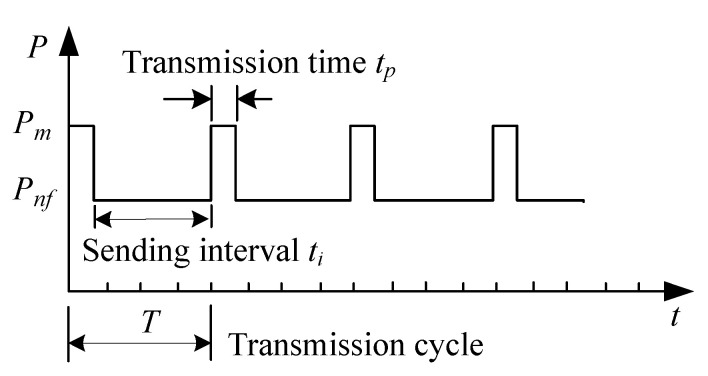
The relationship between transmission time and power consumption.

The transmission duration $t_{p}$ includes measurement preparation time and transmission time. The power consumption is different in these two periods. It is seen from Equations (1) and (2) that the smaller the $t_{p}$, the lower the power consumption. However, there are two constraints in selecting $t_{p}$: the preparation time of measurement and the time required for successful data transmission [24], as illustrated in Figure 14. In this project, a node transmits registration and measurement data once, which includes 16 bytes data (6 bytes of data delimiter, 2 bytes of registration or real-time code, 4 bytes of chip ID, 3 bytes of physical measurements, and 1 byte of cyclic redundancy check value).

The node ID and status information will be recognized and transmitted to the concentrator once, because it is relatively easy to check if a seatbelt is fastened in our monitoring system. However, it is difficult to predict whether the seatbelt is disconnected or not. A timer scheme and a variable counter will be proposed in this work to solve that problem. If the seatbelt is fastened, the counter will become zero, otherwise it will add one unit (for 30 s). If the counter is more than two units, it is assumed that the seatbelt is disconnected. Correspondingly, the error in this monitoring system will be less than 60 s, which is acceptable in real world applications.

### 3.3. Selection of Sleep Mode

**Figure 15:**
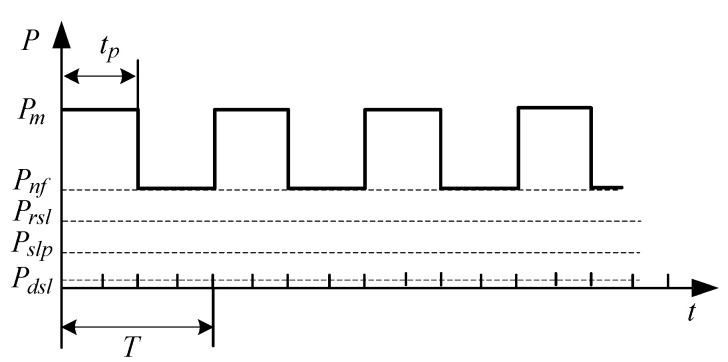
Power consumption corresponding to different working states.

There are five states in each sensor node in our developed monitoring system: operation, standby, sleep-receiving, general sleep, and deep sleep, as summarized Table 1. Figure 15 shows their respective power consumption values $P_{m}$, $P_{nf}$, $P_{rsl}$, $P_{rlp}$, and $P_{dsl}$ in mWh.

**Figure 16:**
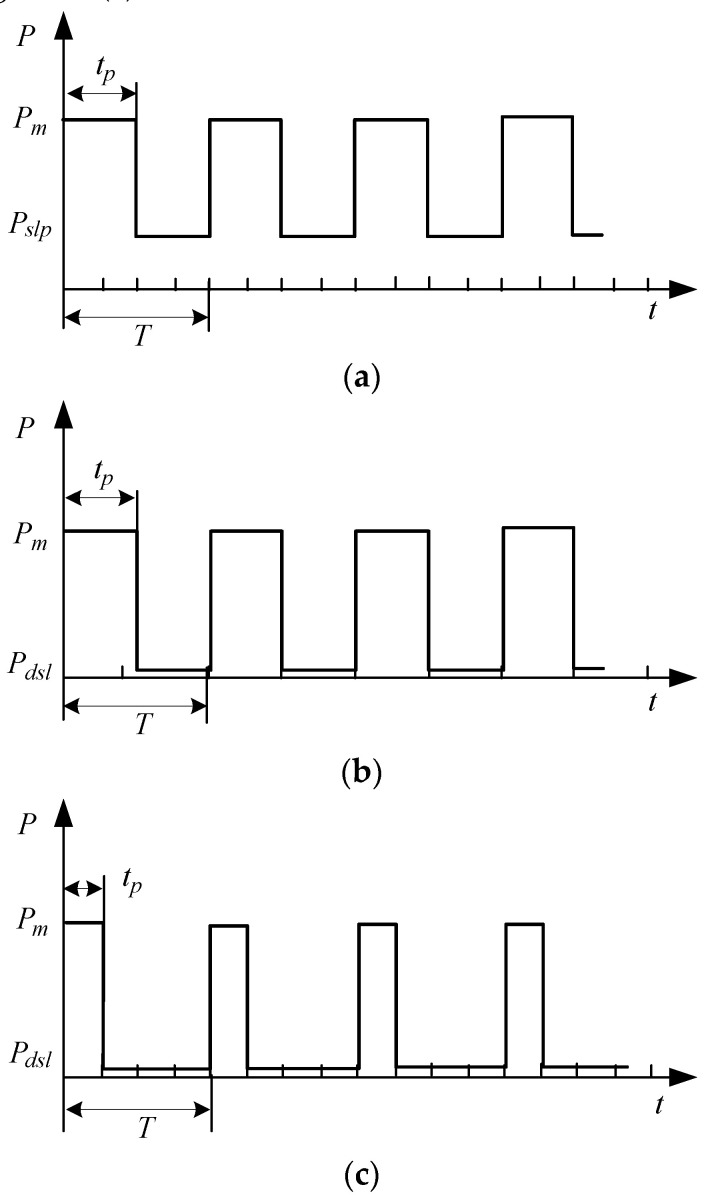
Illustration of power consumption under different sleep modes: (**a**) sleep periodic transmission, (**b**) deep sleep periodic transmission, (**c**) low-power deep sleep periodic transmission.

Different chips could have different sleep modes. In operation, only some of the circuits of the CPU work, and the other circuits may not work. In general sleep, the RF controller of the node works continuously. Once it is called up, the node will be woken up to operate immediately. Deep sleep mode can further reduce power consumption by reducing clock frequency. Figure 16 illustrates some power consumption states of periodic transmission in different sleep modes. The power consumption was the lowest in deep sleep mode. With the same data to be transmitted, shortening the transmission duration $t_{p}$ can reduce the power consumption, especially with the deep sleep periodic transmission mode, as illustrated in Figure 16c.

**Figure 17:**
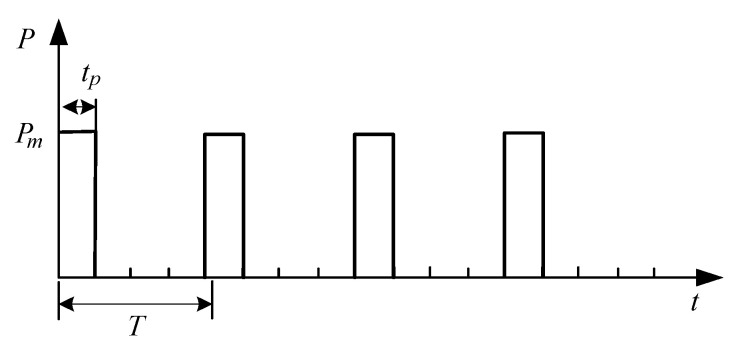
Power consumption of conditional transmission of a node.

In this seatbelt monitoring project, it was assumed that when the data does not change, data processing and transmission are not performed, or the data is transmitted only when the data change occurs in the monitoring system. In conditional transmission as illustrated in Figure 17, a periodic power-on mode will be used; the circuit is not powered on when the seat is not occupied, as illustrated in Equation (2).

## 4. System Performance Testing

Some tests that were undertaken are presented in this section to verify the effectiveness of the proposed power-saving design approaches in the bus seatbelt monitoring system. The tests were performed in two scenarios: system running and node power consumption.

### 4.1. System Running Testing

**Figure 18:**
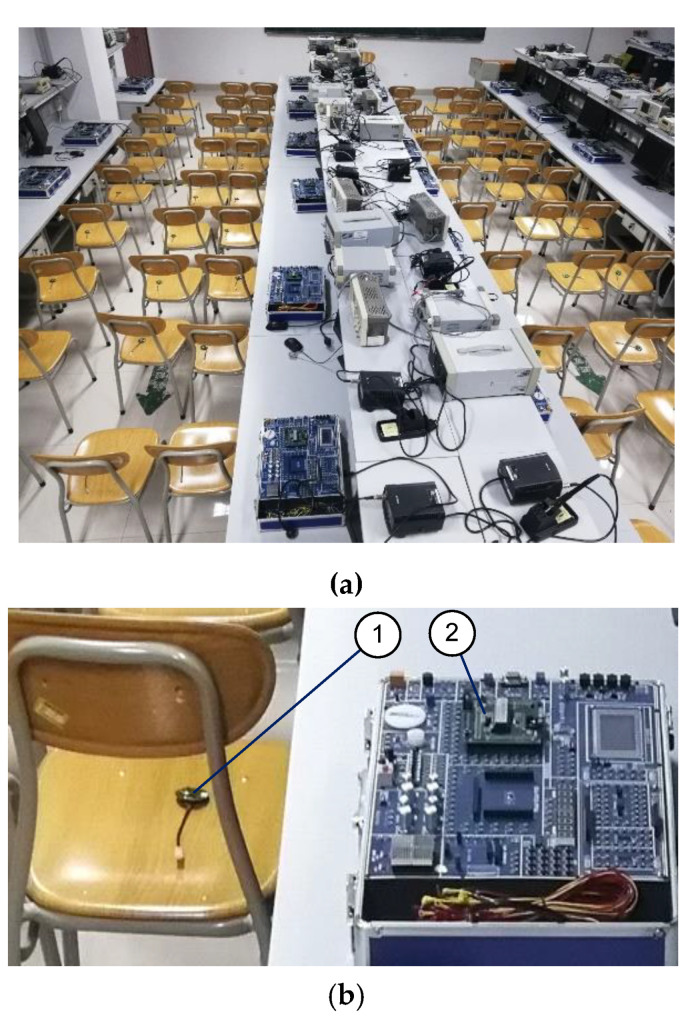
Node test site: (**a**) test site, (**b**) test details: (1) a sensor node, (2) the simulated concentrator unit.

Figure 18a shows the test environment in the lab; the space area was 12 m long and 1.8 m wide, to simulate an intercity bus internal space. Sixty-four chairs were used for testing, each having a sensor node to emulate the seatbelt buckle states. A CAN analyzer was used to examine data integrity. The data from 64 nodes were received by the concentrator simultaneously (only one concentrator was used in this system). The launching distance of each node was about 70 m indoors and 13 m outdoors without data missing and delay, which was appropriate for this bus monitoring application.

**Figure 19:**
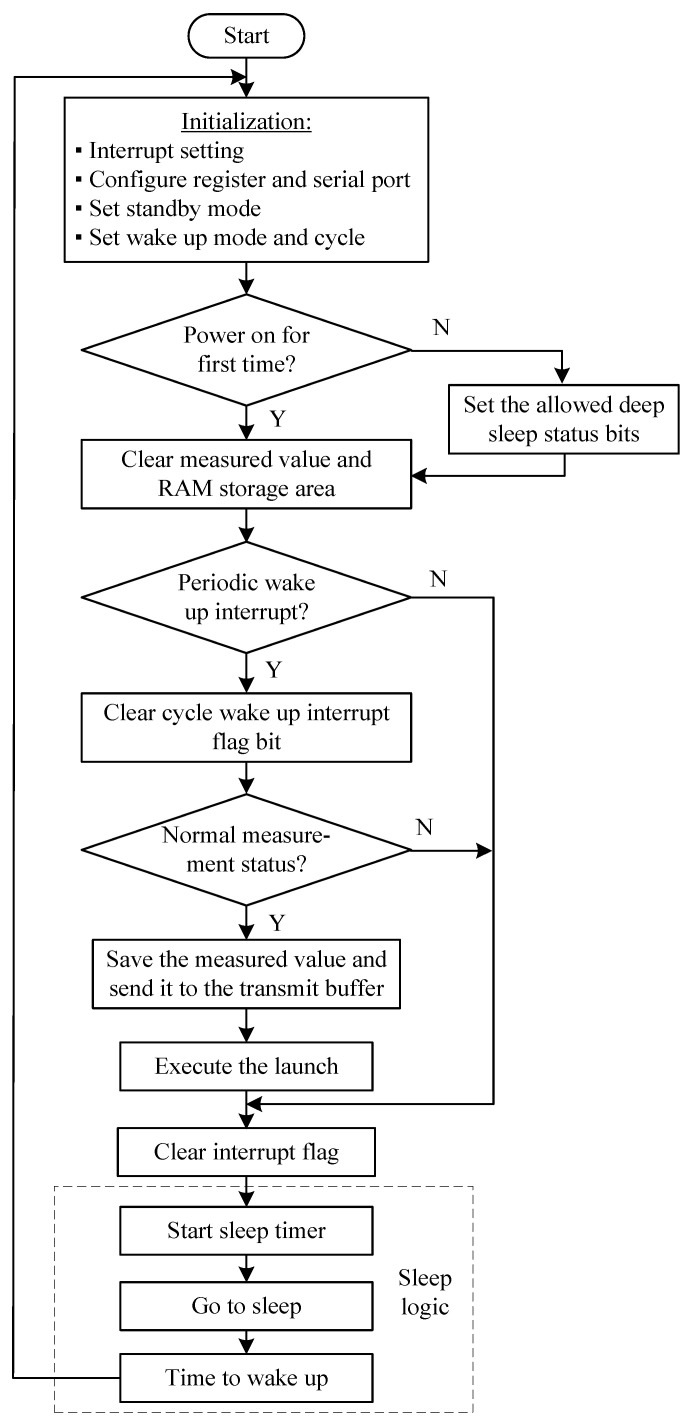
Flow chart of the node power-saving design software.

Figure 19 illustrates processing procedures in power-saving software design. The concentrator received status information from 64 nodes. Then, the MCU detected the deep sleep Power Down Flag (PDF) of the node’s run source. If the PDF was zero, it was to be considered as the first time to power on. The node cleared the interrupt flag bit and set PDF to 1, and made the node turn to the deep sleep mode. The wakeup state was judged by an “AND” operator. When the wakeup duration of 1 min was over, its interrupt flag bit became 1. Then, the node started to measure parameters and sent them out to the concentrator.

### 4.2. Node Battery Current Consumption

Each sensor node was powered by a 3.3-V battery (model: Maxell CR2450HR) with a capability of 550 mAh. The battery capacity *Q* in mAh can be computed by:

(3) $$Q=\frac{Q_{s}+Q_{m}+Q_{x}}{1-T_{a}S_{d}/100}$$

where $Q_{s}$ in mAh is the standby battery capacity; $Q_{m}$ in mAh is the measured battery current consumption; $Q_{x}$ in mAh is the transmission current consumption; $T_{a}$ in hours is the total service life, and $S_{d}$ in % per year is the battery self-discharge rate.

For the periodic transmission mode, the battery current consumption in a sending period $Q_{t}$ can be computed by:

(4) $$Q_{t}=t_{p}I_{Qx}+(T-t_{p})(I_{Qx}+I_{Qm})$$

where $I_{Qx}$ is the current in data transmission; $I_{Qm}$ is the current when the node is in other working modes.

The total service life of the node battery $T_{a}$ will be

(5) $$T_{a}=\frac{100QT}{S_{d}QT+100Q_{t}}$$

**Figure 20:**
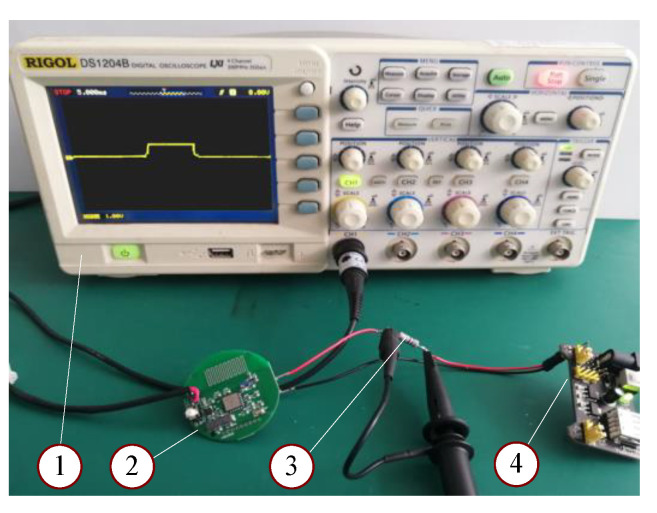
The test system to measure node voltage: (1) oscilloscope, (2) sensor node, (3) a resistor, (4) regulated power supply.

Figure 20 shows the measurement system. The tested battery had self-discharge rate $S_{d}$ = 1% per year. The transmission period *T* was 60 s. Sixteen bytes of data was sent out by each node every cycle with a baud rate of 9600 bits/s. To reduce the voltage drop, a 10-Ω resistor was used in measurement. The voltage drop at both ends of the resistor V(t) was measured using a digital oscilloscope (model: RIGOL DS1204B). Then, the consumption of circuit total current was equal to V(t)/10. Table 2 summarizes the test conditions, and the comparison of cycle current consumption among the general operation design, the general sleep design, and the proposed power-saving deep sleep design. The general sleep design means that the CPU, flash, and RAM were in the hold state, while other parts were off. Deep sleep means that only the LF circuit was on, as listed in Table 1. A serial debugging assistant was used to capture the time of launching 16 bytes of data. The sleeping current was measured using a UA ammeter (with an accuracy ±0.8%), which was put in series to the circuit to measure total current value when the node was in operation, general sleep mode, or deep sleep mode.

In this test, the maximum voltage drop *V*(*t*) = 100 mV, and launching average current was 0.1 V/10 Ω = 10 mA. From Equation (4), the battery current consumption of the power-saving design was $Q_{t}$ = 10 mA × 13 ms + 5.31 uA × 59.987 s = 0.448 mAs. The current of deep sleep power-saving design was 5.31 uA, which is much lower than 56.20 uA in the general sleep design. The average value of sending interval in operation design was 1.86 s, which was measured by using the 433-MHz receiving modular and computer serial debugging assistant.

**Figure 21:**
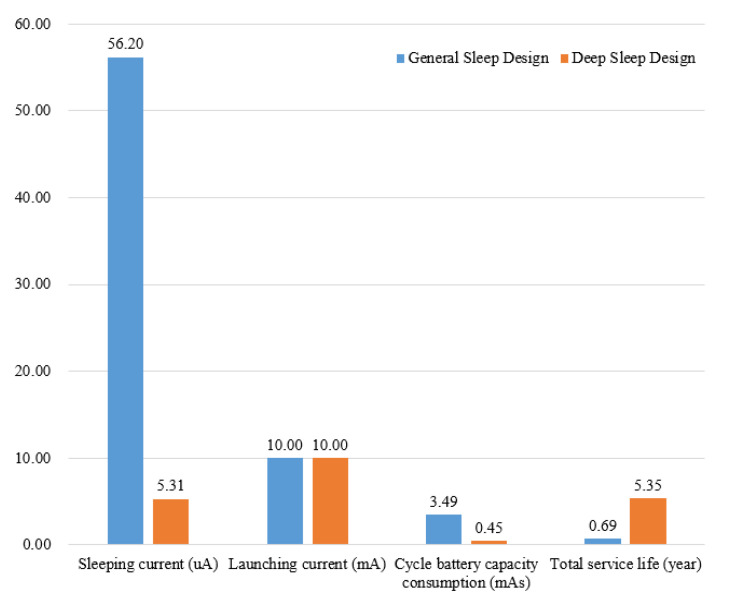
Performance comparison between the general sleep design and the proposed power-saving deep sleep design.

Figure 21 shows the comparison results. From Equation (5), the predicted battery life using the power-saving deep sleep design was about 5.35 years, which was much longer than 0.69 years using the general sleep design strategy. With the same emission current value, the cycle current consumption of the proposed deep sleep power-saving design was 0.45 mAs, which was much smaller than 3.49 mAs by the use of the general sleep design method. The effectiveness of the proposed deep sleep power-saving strategy can then be verified.

## 5. Conclusions

The Internet of Things (IOT) and wireless sensor networks have generated revolutionary impacts in modern industrial applications. In the development of RFID sensor networks, one important issue is related to battery power-saving. This paper investigated power-saving strategies in both hardware and software design. The analysis was based on the development of an intercity bus seatbelt monitoring system. Firstly, a one-to-many pairing node registration scheme was proposed to match the concentrator to the seat nodes to simplify processing and reduce costs. Secondly, a flexible low-power software method was suggested to guide the power-saving design of the RFID network nodes, related to communication mode, transmission cycle, and sleep mode. The effectiveness of the proposed low-power design strategies was verified by some experiments using the developed bus seatbelt monitoring system. Test results showed that in using the deep sleep power-saving method, the cycle current consumption was much lower than using the general sleep mode power-saving design (0.45 mAs vs. 3.49 mAs). Its node service life can be much longer than that using the general saving design (5.35 years vs. 0.69 years). The developed seatbelt monitoring system was successfully used in many intercity buses in China. In addition, the proposed power-saving technique has high potential to be used for more efficient IOT and RFID sensor network applications.

## Figures and Tables

**Table 1:** Node clock allocation for different working modes.

Working Mode	Clock Allocation Description
Operation	Turn on all clock sources. All circuits work normally.
Standby	HF and LF are on. MF is off. CPU and fixed cycle wake-up circuit are in standby state. When the set time is up, CPU is woken up, and enters the operation mode.
Sleep-receiving	MF is on. HF and LF are off. CPU is in sleep mode. RF receiving circuit is always on. Once the request signal of the upper level is detected, MCU will turn into operation mode.
General sleep	LF and MF is on. HF is off. CPU, flash and random-access memory (RAM) are in the hold state. Sensor group can be opened selectively.
Deep sleep	LF is on. MF and HF are off. Only service parameter register, real-time clock and wake-up module are serviced.

**Table 2:** Summary of test conditions and comparison of the battery current consumption.

	Chip Parameters	Operation Design	General Sleep Design	Deep Sleep Design
Maximum launching voltage drop (V)		0.10	0.10	0.10
Launching current (mA)	7.60	10.00	10.00	10.00
Sending interval (s)	59.99	1.86	59.99	59.99
Time of launching 16 bytes (ms)	13.00	14.00	14.10	13.90
Standby/sleeping current (uA)	0.90	95.00	56.20	5.31
Cycle current consumption (mAs)	0.18	581.58	3.49	0.45
Total service life (year)		0.004	0.69	5.35
